# Segmental Agenesis of the Corpus Callosum With Pituitary Hypoplasia

**DOI:** 10.7759/cureus.58727

**Published:** 2024-04-22

**Authors:** Jake Haver, Joseph J Junewick

**Affiliations:** 1 Radiology, Michigan State University College of Human Medicine, Grand Rapids, USA; 2 Radiology, Helen DeVos Children's Hospital, Grand Rapids, USA

**Keywords:** hyposomatotropism, corpus callosum, midline defect, congenital hypopituitarism, septo-optic dysplasia, agenesis of the corpus callosum

## Abstract

We report a 3-year-old male with findings of segmental agenesis of the corpus callosum, pituitary hypoplasia, and Chiari I malformation. The patient was born at 33 weeks and spent five weeks in the NICU for hypoglycemia, hypotension, and dyspnea. In infancy, the patient passed an adrenocorticotropic hormone stimulation test, while cortisol, growth hormone, and insulin-like growth factor levels were within reference range. Following height and weight percentile regression the patient underwent arginine and clonidine stimulation testing at 3 years of age, prompting pituitary evaluation via MRI. The results provided exemplary neuroimaging of segmental callosal agenesis, in which the genu and splenium form despite the absence of the callosal body. This finding adds support to a newer theory of embryological callosal development where progression does not occur linearly in the rosto-caudal direction.

## Introduction

A detectable degree of corpus callosum dysgenesis is found in 1:4000 individuals. Although aberrant callosal variations are not particularly rare, current research still aims at reconciling a mechanism of embryological development. Segmental agenesis of the corpus callosum (segACC) is the segmenting absence of the callosal body despite the development of the genu and splenium. A once-accepted theory of callosal development endorsed a continual rostro-caudal progression from the genu to the splenium, where callosal axons cross through the commissural plate in the superior lamina terminalis, with the cavum septum pellucidum developing from cleavage of the commissural plate [1,2]. This theory has fallen out of favor with support that callosal axons cross the midline through an area of secondary interhemispheric fusion, and that the cavum septum pellucidum is the portion of the interhemispheric fissure that becomes enclosed by the development of the surrounding corpus callosum [1,2]. Support for the latter theory has thus far been predicated upon: 1) Gross examination of anatomic specimens at various stages of development; 2) Magnetic resonance imaging (MRI), as in our case, reveals segmental agenesis between the genu and splenium, suggesting that the midline crossing bed of the developing corpus callosum (week 12 and 13) occurs after the anterior and hippocampal commissures cross (weeks 10 and 11 respectively) [1,2]; 3) Connectivity analysis with diffusion tensor imaging and tractography has revealed that cingulate cortex axonal projections from multiple distinct loci decussate the commissural plate, giving rise to the anterior and hippocampal commissures, which later fuse to form the corpus callosum [1,3]. Although the corpus callosum appears to grow in a rosto-caudal fashion, the commissure actually grows in conjunction with the hemispheres, with the genu developing prenatally, and the splenium mostly growing postnatally [2]. Matter of fact, the corpus callosum does not finish growing until well into the third decade of life [4]. 

Documented conditions associated with callosal agenesis include septo-optic dysplasia (SOD) and Chiari malformations. The patient described in this case was found to have the pituitary hypoplasia characteristic of septo-optic dysplasia, as well as a Chiari I malformation. The clinical presentation of segACC and its associations is highly variable, which is why we delve into the approach providers took in diagnosing this patient. This report highlights the importance of gestationally adjusted laboratory values and proper trending of pituitary hormones and raises awareness of the unique manifestations of callosal agenesis and its associated conditions. Furthermore, this patient's neuroimaging displays the most exemplary account of isolated body segACC to our knowledge, providing robust support for the modern theory of callosal development.

## Case presentation

The patient is a 3-year-old male, born at 33 weeks. Pregnancy was complicated by maternal hypertension and a quarter-pack-per-day cigarette use. Apart from bilateral clubfoot secondary to maternal fibroids, in utero anatomy ultrasounds reported no abnormalities or intracranial processes. Following cesarean delivery, the patient was admitted to the Neonatal Intensive Care Unit (NICU) with increased work of breathing, prolonged hypoglycemia, hypotension, and low free thyroxine with normal thyroid-stimulating hormone levels. 

Further testing revealed an Insulin-like growth factor level of 37 ng/dL (15-150 ng/dL), a random growth hormone level of 0.91 ng/dL (<4.99 ng/dL), and a random cortisol level of 15.1 mcg/dL (3.0-22.0 mcg/dL). Testing excluded congenital adrenal hyperplasia. The patient graduated from the NICU with the resolution of symptoms after 5 weeks.

At 8 weeks of age, the patient was evaluated by endocrinology to clarify the etiology of his previous prolonged hypoglycemia and hypotension. He passed an adrenocorticotropic hormone stimulation test, so further workup was not pursued. At 12 weeks of age, the patient underwent evaluation for stridor, feeding difficulties, and respiratory distress, revealing congenital laryngomalacia - treated with supraglottoplasty, with the resolution of symptoms. At 20 weeks, the patient underwent evaluation for episodic shuddering attacks. The patient's mother reported 30-second long, non-distractible, shuddering episodes that occurred primarily during feeding - neurological examination and electroencephalogram were unremarkable. At 28 weeks, the patient underwent evaluation for vomiting and fussiness, revealing ileo-ileal intussusception - the patient was discharged upon spontaneous resolution, without surgery. At 40 weeks, the patient underwent evaluation for sleep apnea - treated with adenoidectomy, with the resolution of symptoms. At 45 weeks, the patient underwent evaluation for suspected hearing loss due to subjective poor engagement to sound at home, and suspected hearing loss on routine physical examination - a brainstem auditory evoked response test was non-suggestive of hearing loss. At 15 months of age, the patient underwent evaluation for the redevelopment of sleep apnea, revealing pharyngomalacia and tracheomalacia - treated with tonsillectomy, with the resolution of symptoms. From birth until age 2, the patient presented to the emergency department 10 times for prodromal symptoms of viral upper respiratory infection, or gastroenteritis, which would resolve with supportive care.

**Figure 1:**
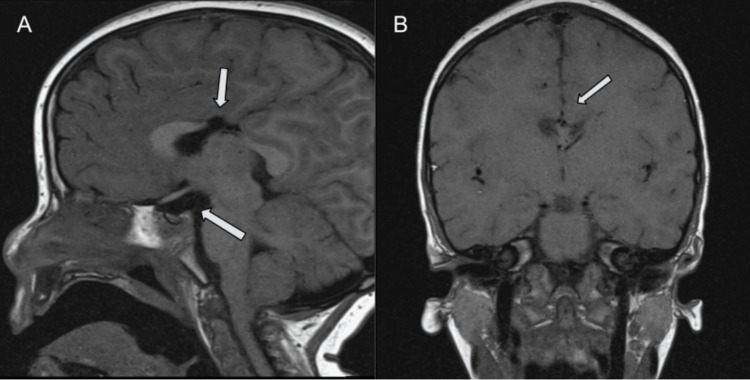
Segmental agenesis of the corpus callosum and pituitary hypoplasia A) Sagittal T1-weighted image (T1WI) shows an intact rostrum, intact genu, absent callosal body, intact isthmus, and intact splenium. Note the small pituitary volume (6.2 x 1.5 x 6.7mm), with a lack of normal T1WI hyperintensity of the neurohypophysis.  B) Coronal T1WI confirms absent commissural fibers bridging the midline.

**Figure 2:**
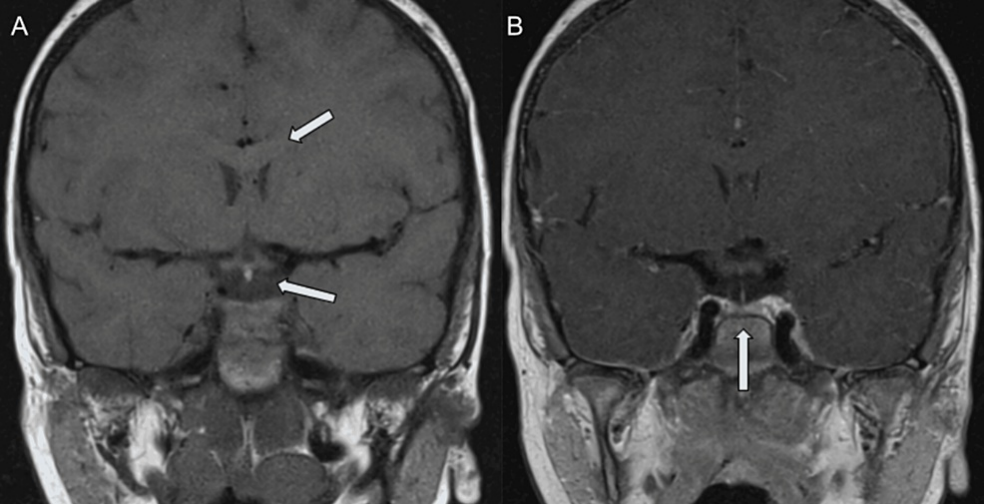
Infundibulum and pituitary hypoplasia on coronal T1 MRI A) Anterior to the callosal body agenesis, T1-weighted image (T1WI) appreciates the presence of commissural genu fibers, and B) a thin infundibulum and hypoplastic pituitary gland.

At 2 years and 11 months of age, the patient underwent evaluation for growth delay. The patient’s height had tracked below the 3rd percentile, and weight had tracked between the 10th and 25th percentiles since birth. Insulin-like growth factor level was now 11 ng/dL (15-150 ng/dL). Growth hormone response was tested via arginine/clonidine stimulation, and revealed severe growth hormone deficiency, prompting MRI evaluation of the pituitary gland. MRI of the brain revealed a Chiari I malformation (6 mm inferior displacement), a small pituitary gland (6.2 x 1.5 x 6.7 mm) with infundibular thinning, and a focally disrupted corpus callosum (Figure 1). Bridging commissural fibers of the genu are present anterior to the absent callosal body (Figure 2). Synthetic growth hormone replacement therapy was initiated.

## Discussion

Connectome and MRI analysis of individuals with segACC has shed light on the various paths axons take when they can not, or do not, properly decussate. Perhaps the most commonly observed surrogate pathway found in segACC would be the formation of Probst bundles - where axons run para-sagittally along the interhemispheric fissure instead of decussating midline [3]. Our case lacks the widely spaced frontal horns, and dilated trigones usually seen with the formation of Probst bundles. 

Attempts have been made to correlate the imaging findings of colossal agenesis with clinical manifestations, but few clinical correlates have been confirmed, and detecting callosal defects is largely incidental. The diagnosis of segACC in our patient was made incidentally after imaging was obtained following the diagnosis of hyposomatotropism post arginine and clonidine stimulation test. In retrospect, the patient’s hypotension, low thyroxine, hypoglycemia, growth delay, and normal adrenocorticotropic hormone stimulation test may make a pituitary etiology seem overt, though cortisol, growth hormone, and insulin-like growth factor levels remained within reference range on testing until significant growth delay was observed. Consequently, diagnosis of a hypoplastic pituitary gland, and initiation of replacement growth hormone, was not made until nearly 3 years of age. This brings up the point that without gestationally appropriate reference intervals, the sampling of random and isolated pituitary hormone levels is often futile for diagnostic purposes [5]. This makes appropriate trending of growth hormone and insulin-like growth factors necessary in apparently symptomatic infants. 

Since the advent of more advanced imaging techniques, the association between structural midline defects and hypopituitarism has been increasingly documented in the literature. Midline defects in the septum pellucidum or corpus callosum, and pituitary hypoplasia/deficiency are two of the three characteristic findings in the common (~1:10,000) condition SOD, with optic nerve hypoplasia completing the triad. Two of the three aforementioned findings are usually sufficient for diagnosis, as only 30% of patients with SOD will present with the complete triad [6]. Therefore, our patient with segACC and hyposomatotropism, but without optic nerve findings, still fits the criteria for SOD. The septum pellucidum was also normal in this patient. It should be noted that SOD is a phenotypic manifestation of numerous identified genotypes, therefore there is low specificity in genetic testing, with a known defect detection rate being under 10% [6,7].

## Conclusions

This case of a 3-year-old male with segmental agenesis of the corpus callosum adds validity to an increasingly supported theory that embryological development of the corpus callosum results from secondary midline fusion from multiple distinct loci, rather than a continuous rosto-caudal progression. Furthermore, it endorses the association between midline commissure defects and pituitary hypoplasia.
